# Moving Beyond Screening: How Emergency Departments Can Help Extinguish the HIV/AIDS Epidemic

**DOI:** 10.5811/westjem.2016.1.29100

**Published:** 2016-03-02

**Authors:** Michael Menchine, Michael Zhou, Shahram Lotfipour, Bharath Chakravarthy

**Affiliations:** * Keck School of Medicine of USC, Department of Emergency Medicine, Los Angeles, California; † USC Schaeffer Center for Health Policy and Economics, Los Angeles, California; ‡ Tufts University School of Medicine, Boston, Massachusetts; § University of California Irvine School of Medicine, Department of Emergency Medicine, Irvine, California

## Abstract

While great strides have been made in diagnostic and treatment strategies, human immunodeficiency virus (HIV) remains a major public health epidemic. The Centers for Disease Control and Prevention (CDC) Morbidity and Mortality Weekly Report article, “Vital Signs: HIV Diagnosis, Care, and Treatment Among Persons Living with HIV – United States, 2011,” highlights current areas of concern regarding HIV diagnosis and care. The CDC estimates that 1.2 million people in the U.S. are living with HIV. Of them, 86% have received a diagnosis (14% remain undiagnosed and unaware), but only 40% are engaged in care and a mere 30% are virally suppressed. Emergency departments (EDs) can play a major role in combatting the HIV epidemic through regular screening and facilitating linkage to chronic HIV care. Universal opt-out screening as recommended by the CDC in 2006 has been shown to be effective but expensive, and has not been widely implemented in EDs nationwide. Cost-effective models and a renewed commitment from ED providers are needed to enhance ED-based HIV containment strategies.

## CDC MORBIDITY AND MORTALITY WEEKLY REPORT FINDINGS

In the November 28, 2014 issue of the Morbidity and Mortality Weekly Report (MMWR), the Centers for Disease Control and Prevention (CDC) published data and trends concerning human immunodeficiency virus (HIV) diagnosis and care. The report demonstrates that, despite advances in medical therapy that make HIV highly manageable, the proportion of individuals living with HIV who are virally suppressed is unacceptably low.

The CDC found that in 2011, an estimated 1.2 million individuals were living with HIV in the United States. Of those, 86% had been diagnosed, but only 40% were engaged in care, 37% were prescribed antiretroviral therapy (ART), and 30% had achieved viral suppression. Of the 70% who had not achieved viral suppression, 20% were unaware of their HIV status, 66% were diagnosed but were never engaged in care, 4% received care but were never prescribed ART, and 10% were prescribed ART but never achieved viral suppression. Viral suppression was similarly poor among Blacks and Whites but significantly lower in younger adults compared with older individuals.

The MMWR article also reported that of the newly diagnosed individuals in 2011, only 80% were linked to medical care within three months. Even lower linkage rates were observed among younger individuals (73% for those 13–24), and Blacks (76%). Of those who did engage in HIV treatment, 92% were prescribed ART and 76% achieved viral suppression. ART has been shown to decrease the rate of HIV transmission by 96% and dramatically increase life expectancy. An individual diagnosed with HIV at age 20 who immediately starts and continues ART treatment can be expected to live to age 71, approaching the average lifespan of 79. An individual diagnosed at age 20 who does not initiate ART treatment is expected to live only to age 32.^1^

## COMMENTARY

Advances in HIV treatment have transformed the natural history of this illness from one of near-certain death within a decade to one best conceptualized through a chronic care model. So effective are the treatment options that some have audaciously challenged the healthcare system to produce an “Acquired immunodeficiency syndrome (AIDS)-Free Generation,”^2^ Although ambitious, we may well have the medical treatments needed to fulfill this challenge. In order for one to be virally suppressed, multiple barriers must be overcome. First, the person must be tested and diagnosed with HIV. Next, he or she must be linked to an HIV care provider and be prescribed ART. Finally the patient must be adherent with medications and routinely monitored for viral suppression. In practice, patients fall off at each step of this “HIV Care Continuum.”^3^

In their role as safety net providers, emergency departments (EDs) play a key role in both the diagnosis of HIV and linkage to HIV-specific care. HIV infection is particularly well-suited to screening efforts because 1) it is life-threatening and can be detected long before symptoms develop; 2) rapid, non-invasive and inexpensive tests are available for early detection;^4^ 3) if diagnosed and treated, infected people gain decades of life expectancy;^1^ 4) mere identification of HIV-infected individuals dramatically reduces risk behaviors and transmission rates;^5^ 5) the cost-effectiveness of treatment has been proven;^6^ and 6) EDs serve a safety net population that has a high burden of undiagnosed infection and are unlikely to be screened elsewhere.^7^

These features that would tend to favor HIV screening in the ED have been undercut by cost concerns, legal requirements for consent and, perhaps most importantly, social stigma. Stigma surrounding HIV create an impression that HIV infection marks an individual’s failure to adhere to sex or drug norms and directly results in shame, embarrassment and isolation. Fear of these stigma causes at-risk people to shun healthcare professionals, lie about risk factors and avoid HIV testing and/or information.^8^ For many the stigma of HIV makes asking for the test prohibitive.^9^ The World Health Organization cites stigma as a dominant reason for not being tested, not lack of access to the test.^10^ These stigma impact physicians as well who may be reticent to offer HIV testing so as not to offend their patients.^11^ These issues coupled with legal requirements that demanded significant pre- and post-test counseling made HIV testing in a time-limited setting such as an ED infeasible.

In 2006, the CDC acted to counter these prevailing barriers to screening by calling for non-targeted, “opt-out” HIV screening for all patients aged 13–64 in all settings where the prevalence of undiagnosed HIV was greater than 1 in 1000. (Nearly all EDs meet this threshold.) In calling for non-targeted screening, the CDC was attempting, in part, to remove the embarrassment/stigma associated with asking to provide or receive the test. The proposed mantra became “we test everyone” regardless of risk and being offered the test does not label the patient as “high risk.” The CDC further reduced HIV “exceptionalism” by recommending opt-out testing that does not require separate written consent – putting HIV testing in line with almost all other testing practices.^12^

In general, albeit slowly, state legislatures have followed the CDC recommendations, and most states have removed special written consent for HIV testing. California, for example requires only a verbal opt-out consent process that essentially amounts to telling the patient he or she is going to be tested and conducting the test, unless the person specifically declines. Specific counseling or documentation of test acceptance is not required. In such a context, opt-out screening is accepted by over 90% of patients.^13^ However, a positive result can cause significant emotional distress, and such results should be disclosed professionally and with psychological and social assistance available.

Despite these powerful guidelines and legal changes, EDs have been slow to implement routine testing. A 2009 survey found that though 82% of EDs offer some type of HIV testing, only 22% do so in a systematic way, and merely 7% do so using the suggested “opt-out” model.^14^ Now, the greatest barrier to providing optimal HIV screening cited by EDs is cost.^15^ Indeed, the ideal cost model is a matter of significant debate. One estimate shows that universal opt-out screening results in costs of $9,900 per new HIV diagnosis. Though cost-effective by traditional standards (e.g. <$50,000 per quality-adjusted life year saved), this is nonetheless an expensive strategy.^16^ Another proposed model uses the Denver HIV risk score. The Denver HIV risk score stratifies patients into risk groups based on demographic characteristics and risk behaviors (which are obtained by structured interview), and diagnostic HIV tests are only performed on those at higher risk. Preliminary data show that this method can detect similar numbers of HIV infections with fewer tests and up to 20% lower costs ($7,800 per new HIV diagnosis) compared to a more universal opt-out strategy.^17^ A large multicenter clinical trial is currently underway to test the cost and effectiveness of the two strategies head-to-head. Regardless of which approach prevails, it bears emphasizing that both require screening all patients for HIV. The Denver score simply uses the risk tool as the initial screen and relies less on diagnostic testing, while the universal, opt-out approach relies solely on diagnostic testing.

While diagnosis is a critical first step towards virologic suppression and controlling further infection (once a person is diagnosed with HIV, risk behaviors drop precipitously),^5^ it is not sufficient. Linking newly diagnosed patients to care is vital and has been proven to be feasible.^18^–^20^ The precise linkage team composition and services provided will depend on individual ED volume and resources but may include nurses, physicians, case-managers, social workers and/or patient navigators. Ultimately, this team helps deliver positive test results, provides HIV counseling, offers case management services, assists with transportation, provides social services and facilitates linkage to care with the goal of seeing a provider who can prescribe ART.^19^ In fact, designated HIV linkage teams consisting of a nurse practitioner, registered nurse, and social worker successfully linked 93.9% of newly diagnosed patients to long-term care.^20^

Although finding undiagnosed cases remains a centerpiece of combating the HIV epidemic, the overwhelming majority of people with uncontrolled HIV infection are well aware of their condition.^1^ ED-based screening programs note that the majority of patients who have a positive HIV test were likewise aware of their HIV status and many were recognized by the treating physicians as having HIV.^21^ HIV is a common chronic illnesses seen in the ED (up to 7.8% of the ED population).^21^ Seemingly, repeating the HIV test in these circumstances adds little, and efforts to eliminate these wasteful tests should be pursued. However, an additional benefit of opt-out screening with a robust linkage team is that it can be used to re-link known HIV positive individuals who have fallen out of care. In one of the largest ED-based HIV screening programs in the country, investigators in Houston demonstrated that the linkage strategies associated with their opt-out screening program boosted engagement in care from 41.3% to 58.8% among patients with known HIV infection. Retention in care and virologic suppression were likewise substantially enhanced (from 32.6% to 47.1% and 22.8% to 34% respectively). The effect was most pronounced among younger patients (age 16–24) who saw retention in care increase from 15% to 37%.^19^ So, while efforts to minimize redundant HIV testing among those known to be HIV positive should be pursued, so should efforts to ensure that those with chronic HIV infection are appropriately linked to care.

Thirty years into the epidemic, HIV is now unquestionably a manageable, chronic disease. However, despite our ability to manage the illness, only 30% of those infected are virologically suppressed and 14% are unaware of their infection.^1^ EDs are often the primary or sole healthcare provider for patients with chronic uncontrolled HIV and treat a patient population with a high prevalence of undiagnosed HIV and, as such, must be part of a comprehensive solution to the epidemic. Many questions remain open: Should universal screening be based solely on diagnostic testing or some combination of risk assessment and testing? What is the most cost-effective formulation of a linkage team? How can we best leverage health records to reduce duplicate testing while still targeting out-of-care individuals? What payment models will sustain these efforts? Research is ongoing to answer each of these questions. What can no longer be in question, however, is the need for EDs throughout the nation, particularly those that espouse to serve as safety net providers for vulnerable people, to be, at a minimum, willing partners and, on occasion, leaders in the audacious pursuit of an AIDS-free generation.
